# Ionic Gelation Controlled Drug Delivery Systems for Gastric-Mucoadhesive Microcapsules of Captopril

**DOI:** 10.4103/0250-474X.45410

**Published:** 2008

**Authors:** M. A. Altaf, Sreedharan, N. Charyulu

**Affiliations:** Department of Pharmaceutics, N. G. S. M Institute of Pharmaceutical Sciences, Mangalore-575 005, India

**Keywords:** Captopril, controlled release, ionic gelation, microcapsules, mucoadhesion and oral drug delivery systems, sodium alginate

## Abstract

A new oral drug delivery system was developed utilizing both the concepts of controlled release and mucoadhesiveness, in order to obtain a unique drug delivery system which could remain in stomach and control the drug release for longer period of time. Captopril microcapsules were prepared with a coat consisting of alginate and a mucoadhesive polymer such as hydroxy propyl methyl cellulose, carbopol 934p, chitosan and cellulose acetate phthalate using emulsification ionic gelation process. The resulting microcapsules were discrete, large, spherical and free flowing. Microencapsulation efficiency was 41.7-89.7% and high percentage efficiency was observed with (9:1) alginate-chitosan microcapsules. All alginate-carbopol 934p microcapsules exhibited good mucoadhesive property in the *in vitro* wash off test. Drug release pattern for all formulation in 0.1 N HCl (pH 1.2) was diffusion controlled, gradually over 8 h and followed zero order kinetics.

Microencapsulation by various polymers and their applications are described in standard textbook^1^. Microencapsulation and the resulting microcapsules have gained good acceptance as a process to achieve controlled release and drug targeting. Mucoadhesive is a topic of current interest in the design of drug delivery systems to prolong the residence time of the dosage form at the site of application or absorption and to facilitate intimate contact of the dosage form with the underlying absorption surface to improve and enhance the bioavailability of drug^2^. This study describes the formulation and evaluation of gastric-mucoadhesive microcapsules of captopril employing various mucoadhesive polymers designed for oral controlled release. Captopril is an angiotensin converting enzyme inhibitor used in the treatment of hypertension and congestive cardiac failure, which requires controlled release owing to its short biological half-life of 3 h and the drug, is unstable in the alkaline pH of the intestine, where as stable in acidic pH and specifically absorbed from the stomach^3^,^4^.

Based on the above reasons there is a clear need to localize the developed formulation at the target area of GIT. Microcapsules containing captopril were prepared employing sodium alginate in combination with four mucoadhesive polymers like hydroxypropylmethylcellulose, carbopol 934p, cellulose acetate phthalate and chitosan. Emulsification-ionic gelation process was used to prepare the microcapsules^4^.

Core coating material (sodium alginate) and the mucoadhesive polymers were dissolved in distilled water (32 ml) to form a homogeneous polymer solution. Core material (captopril 1 g) was added to the polymer solution and mixed thoroughly to form a smooth viscous dispersion. The resulting dispersion was then added in a thin stream to a 300 ml of arachis oil contained in a 500 ml beaker with stirring at 400 rpm using a mechanical stirrer. The stirring was continued for 5 min to emulsify the added dispersion as fine droplets. Calcium chloride (10% w/v) solution (40 ml) was then added slowly while stirring for ionic gelation (or curing) reaction. Stirring was continued for 15 min to complete the curing reaction and to produce spherical microcapsules. Mixture was then centrifuged and the product thus separated was washed repeatedly with water and dried at 45° for 12 h. Based on the above procedure various formulations were developed as given in Table 1

**TABLE 1:** COMPOSITION AND CHARACTERISTICS OF CAPTOPRIL GASTRIC-MUCOADHESIVE MICROCAPSULES

Composition in g	Formulation code and ratio of Polymers used							

	(1:1)				(9:1)			
		
	F1	F2	F3	F4	F5	F6	F7	F8
Captopril	2.0	2.0	2.0	2.0	2.0	2.0	2.0	2.0
Alginate	1.0	1.0	1.0	1.0	2.7	2.7	2.7	2.7
Hydroxy propyl	1.0	--	--	--	0.3	--	--	--
methyl cellulose								
Carbopol 934p	--	1.0	--	--	--	0.3	--	--
Chitosan	--	--	1.0	--	--	--	0.3	--
Cellulose acetate	--	--	--	1.0	--	--	--	0.3
phthalate								
Physicochemical property								
Percentage yield (%)	67.0± 5.06	52.6± 7.08	69.1± 09.4	52.40± 09.4	52.55± 8.07	80.58± 05.5	68.78± 09.8	79.55± 06.4
Viscosity (CPS)	7650	8150	7450	7250	7750	7501	7200	7915
Drug content (mg)	1.14± 0.051	1.05± 0.044	1.27± 0.073	0.86± 0.026	1.6± 0.047	1.72 ± 0.036	1.81± 0.018	1.4± 0.019
Average diameter (μm)	40.5± 4.6	48.56± 08.2	45.1± 1.5	40.7± 5.09	51.6± 7.06	65.0± 1.5	62.8± 4.60	52.2± 7.0
Microencapsulation	57.0	52.5	63.5	41.7	68.0	71.5	79.0	60.7
efficiency (%)								
Mucoadhesion (%)	44.0±1.0	62.0±1.52	12.0±0.30	16.0±0.45	22.0±1.65	54.0±1.91	12.5±0.48	8.2±0.21
Swelling and adhesion	++	++	+	+	+	++	+	+
Stability	++	++	++	++	++	++	++	++

The value indicates Mean±SD. Standard deviation is between n = 8 ++ good adhesion, + fair adhesion. The value indicates Mean±SD. Standard deviation is between n = 8 ++ good stability.

Drug content estimation was done by a reported method by El-Kamel *et al*.^5^, 20 mg of the microcapsules were stirred in 3 ml of sodium citrate solution (1% w/v) until complete dissolution occurs. One milliliter of methanol was added to sodium citrate solution to gel the solubilized calcium alginate and further solubilise captopril. This solution was then filtered to obtain drug solution. The filtrate is suitably diluted with 0.1N HCl and absorbance was taken at 212 nm.

Microencapsulating efficiency was calculated using the formula; microencapsulation efficiency is equal to the ratio of estimated percentage of drug content by theoretical percentage of drug content into 100. Dissolution studies were performed for microcapsules containing quantity equivalent to 100 mg of drug filled in capsules by using USP 23 TDT-06T (Electro lab-paddle method) at 50 rpm. The media used were 900 ml of 0.1 N HCl (pH 1.2), maintained at 37±0.5°, 5 ml of samples were withdrawn at different time intervals and replace with 5 ml of dissolution medium. The samples were filtered and assayed spectrophotometrically at 212 nm^6^–^8^, after appropriate dilutions. Dissolution testing was also performed for 100 mg pure drug.

The mucoadhesive property of the microcapsules was evaluated by an *in vitro* adhesion testing method, known as wash off method. The mucoadhesive property of microcapsules was compared with that of a non-adhesive material, ethylene vinyl acetate microcapsules. A piece of sheep stomach mucosa (2×2 cm) was mounted onto glass slide (3×1 inch) with cyanoacrylate glue. One more glass slides were connected with a support. Fifty microcapsules were counted and spread over the wet rinsed tissue specimen and immediately there after the support was hung on the arm of a USP tablet disintegrating test machine^9^,^10^. By operating the disintegration machine the tissue specimen was given a slow regular up and down moment. The slides move up and down in the test fluid at 37±0.5°. The number of microcapsules adhering to the tissue was counted at 2 h intervals up to 8 h. The test was performed in acidic media (0.1N HCl pH 1.2).

Microcapsules of captopril with a coat consisting of alginate and a mucoadhesive polymer (1:1) and (9:1) ratio namely hydroxy propyl methyl cellulose, carbopol 934p, chitosan and cellulose acetate phthalate could be prepared by emulsification ionic gelation process. The prepared microcapsules were found to be discrete, large, spherical and free flowing. The size of all the formulated microcapsules was found to be in the range of 40.5 μm to 65 μm.

Based on the amount of drug loaded in microcapsules, microencapsulation efficiency was calculated. There was no significant difference observed in drug loading. As the proportion of alginate was raised from 1 to 9, there was significant increase in the microencapsulation efficiency of microcapsules.

*In vitro* wash off test was performed to understand the mucoadhesive property of microcapsules. Here the time of detachment of microcapsules from sheep stomach mucosa was measured in 0.1N HCl (pH 1.2). All the formulated microcapsules demonstrated good mucoadhesive property compare to non-mucoadhesive polymer (ethylene vinyl acetate). The following stages have occurred during mucoadhesion. Initially, an intimate contact between the mucus gel and the swelling of mucoadhesive polymer that is (wetting), which makes the polymer strands to relax this is followed by the penetration of the mucoadhesive polymer into the mucus gel network and finally the formation of secondary chemical bonds between the mucus and the mucoadhesive polymer^11^,^12^.

*In vitro* release studies were carried out in 0.1 N HCl (pH 1.2) which indicated that there was a slow and controlled release of drug for all the formulations (Figs. 1 and 2). Alginate-cellulose acetate phthalate microcapsules demonstrated sustained release compared to all other alginate polymer combinations. The order of drug release was found to be zero order for all the formulations. Drug release data was better fit to Higuchi's diffusion model and the release of drug from all the formulations is diffusion rate limited^13^. The order of increasing release rate observed with microcapsules was alginate-cellulose acetate phthalate microcapsules < alginate-chitosan microcapsules < alginate-carbopol 934p microcapsules < alginate-hydroxypropylmethylcellulose microcapsules.

**Fig. 1:**
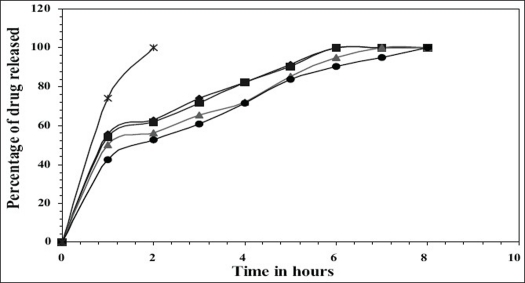
Comparative *in vitro* release profiles of captopril from (1:1) ratio of Polymers used formulations *In vitro* cumulative release of captopril from F1 (-♦-); F2 (-▪-); F3 (-▲-); F4 (-●-); Pure drug (-*-).

**Fig. 2:**
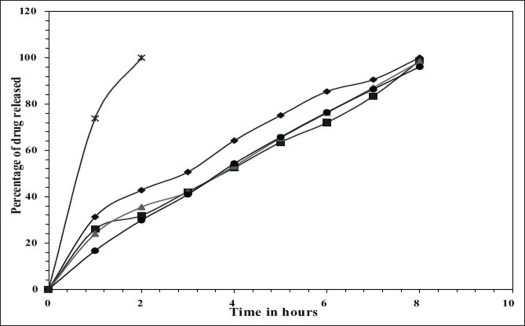
Comparative *in vitro* release profiles of captopril from (9:1) ratio of Polymers used formulations *In vitro* cumulative release of captopril from F5 (-♦-); F6 (-▪-); F7 (-▲-); F8 (-●-); Pure drug (-*-).

Thus, large sized spherical microcapsules with a coat consisting of alginate and a mucoadhesive polymer (hydroxypropylmethylcellulose, Carbopol 934p, Chitosan and cellulose acetate phthalate) could be prepared by emulsification ionic gelation process. The microcapsules exhibited good mucoadhesive property *in vitro*-wash off tests. Captopril releases from these mucoadhesive microcapsules slow and extended over longer period of time. Drug release was diffusion controlled and followed zero order kinetics. Alginate-Carbopol 934p microcapsules and alginate-chitosan microcapsules were found suitable for oral controlled release.
